# The Comparison between the Composition of 100% Autologous Serum and 100% Platelet-Rich Plasma Eye Drops and Their Impact on the Treatment Effectiveness of Dry Eye Disease in Primary Sjogren Syndrome

**DOI:** 10.3390/jcm12093126

**Published:** 2023-04-25

**Authors:** Dominika Wróbel-Dudzińska, Agata Przekora, Paulina Kazimierczak, Agnieszka Ćwiklińska-Haszcz, Ewa Kosior-Jarecka, Tomasz Żarnowski

**Affiliations:** 1 Department of Diagnostics and Microsurgery of Glaucoma, Medical University of Lublin, 20-059 Lublin, Poland; 2 Independent Unit of Tissue Engineering and Regenerative Medicine, Medical University of Lublin, 20-059 Lublin, Poland

**Keywords:** dry eye disease, primary Sjogren syndrome, growth factors, autologous serum platelet-rich plasma

## Abstract

Purpose: The aim of the study was to compare the difference in composition between 100% autologous serum (AS) and 100% platelet-rich plasma (PRP) eye drops and assess their impact on the clinical outcomes after the treatment of severe dry eye (DE) in primary Sjogren Syndrome patients (pSS). Materials and Methods: This is an interventional, non-randomized, comparative, three-month study. 22 patients with severe DE in pSS were treated with 100% AS (22 eyes) and 100% PRP (22 eyes) eye drops 5 times per day in monotherapy mode. The quantifications of growth factors (GFs) such as fibroblast growth factor (FGF), epidermal growth factor (EGF), vascular endothelial growth factor (VEGF), platelet-derived growth factor (PDGF), nerve growth factor (NGF), transforming growth factor (TGF-b), insulin-like growth factor (IGF), fibronectin, and substance *p* in hemoderivates were done. The main outcome measures were: Ocular Surface Disease Index (OSDI), Best Corrected Visual Acuity (BCVA), the Schirmer test, tear break-up time (TBUT), corneal and conjunctival staining according to the Oxford scale, conjunctival hyperaemia, and Meibomian gland parameters. The results were compared at baseline, 1 month, and 3 months following the treatment. The clinical results were correlated with the concentration of GFs in the biological tear substitutes. Results: Significant differences were observed in the concentration of FGF (4.42 ± 0.86 vs. 15.96 ± 7.63, *p* < 0.0001), EGF (4.98 ± 0.97 vs. 39.06 ± 20.18, *p* < 0.0001), fibronectin (929.6 ± 111.5 vs. 823.64 ± 98.49, *p* = 0.0005), VEGF (175.45 ± 65.93 vs. 717.35 ± 488.15, *p* < 0.0001), PDGF AB (619.6 ± 117.30 vs. 349.66 ± 79.82, *p* < 0.0001), NGF (85.22 ± 23.49 vs. 8.29 ± 9.06, *p* < 0.0001), PDGF (935.38 ± 434.26 vs. 126.66 ± 54.41, *p* < 0.0001), substance *p* (112.58 ± 27.28 vs. 127.51 ± 26.56, *p* = 0.0125) in PRP compared to AS. The level of TGF-β was undoubtedly higher in AS than in PRP (1031.37 ± 330.23 vs. 726.03 ± 298.95, *p* = 0.0004). No significant differences between AS and PRP were observed in the concentration of IGF. Therapy with blood products relieved the signs and symptoms in pSS DE patients. There was a statistically significant improvement in BCVA, the Schirmer test, TBUT, Meibomian gland parameters, and the reduction of the OSDI scores, Oxford staining, and conjunctiva hyperaemia in each of the groups. However, the clinical changes were more significant in the PRP group. There were numerous correlations between the level of GFs and the mean change in clinical outcomes. No adverse events were reported. Conclusions: Despite the fact that blood derivatives differ in composition, they seem to be effective and safe in the treatment of severe DE in pSS patients. The signs and symptoms of DE were reduced in both groups, but only the mean change in OSDI was statistically significant. A greater reduction in OSDI scores was observed in the PRP group. The obtained results and the composition of haemoderivates may indicate the superiority of PRP in relieving the symptoms of DE in pSS patients compared to AS.

## 1. Introduction

Sjogren’s syndrome (SS) is a complex autoimmune disease with multiple lesions resulting. The main manifestations include mouth and eye dryness, accompanied by systemic complications (e.g., lymphoma, pulmonary disease, kidney injury, and central or peripheral nervous system neuropathies) [1]. KJ Bloch introduced the concept of primary Sjogren syndrome (pSS), where there is no underlying cause for any rheumatic disorder, and secondary Sjogren syndrome (sSS), which coexists with an independent connective tissue disease (rheumatoid arthritis, systemic lupus erythematosus, scleroderma, dermatomyosis, primary ciliary cirrhosis) [2]. Its prevalence ranges from 0.5 to 1% among the general population [3]. SS mainly affects middle-aged women, with a male-to-female ratio of 1:9 [4]. Dry eye (DE) might be the first clinical manifestation of Sjogren syndrome (SS), which primarily targets exocrine glands, including the salivary and lacrimal glands. The destruction of the lacrimal gland mediated by T lymphocytes leads to the development of an aqueous-deficient tear film. Moreover, this constitutes a trigger factor for the vicious and self-perpetuating cycle of DE with tear film hyperosmolarity and inflammation [5].

Ocular or oral dryness is present in approximately 95–98% of pSS patients, and as many as 89% of patients present with both [6]. Ocular symptoms are as follows: blurred vision, eye irritation, foreign body sensation, and photophobia. Untreated DE might evolve into neurotrophic keratitis, thinning and corneal melting, ulceration, and perforation. Thus, treatment is extremely important to avoid vision-threatening complications. Furthermore, the signs and symptoms of DE may negatively affect daily activities, influencing the patient’s quality of life.

Due to the multifactorial etiology, the treatment poses a challenge and presents limited efficacy. Current management of Sjogren syndrome dry eye (SSDE) includes topical administration of artificial tear substitutes such as drops, gels, or ointments whose task is lubrication of the ocular surface. Unfortunately, the substitutes lack ingredients found in natural tears, e.g., proteins, lipids, and growth factors. Moreover, in moderate or severe DE, only preservative-free artificial tears should be considered due to the harmful, toxic, or allergic effects of the common-use preservatives on the already damaged ocular surface. Considering the accompanying inflammation process, other therapeutic options are glucocorticoids and cyclosporine A, but their side effects (intraocular pressure increase, glaucoma, irritation) should not be ignored. The next step of the DE treatment is interventional procedures such as punctal occlusion, amniotic membrane transplantation, or the application of neurostimulators. Due to the limited success of artificial tears [7] (lack of the components of natural tears) as well as some adverse effects and their high cost, researchers started looking for a new alternative, a personalized treatment. Encouraged by the positive results of blood derivative application in other specialties, ophthalmologists started to search for the possibility of autologous hemoderivate drop application for ocular surface treatment.

Blood-derived eye drops have recently become more popular due to their properties mimicking the components of natural tears. They are indicated as a third-line option for severe dry eye disease, according to the TFOS II Report [8]. Blood-derived eye drops such as autologous serum (AS) or platelet-rich plasma (PRP) are composed of nutrients and growth factors that are unavailable in artificial tears. Those biological tear substitutes contain substances P, fibronectin, platelet-derived growth factor, nerve growth factor, epidermal growth factor, and transforming growth factor-β, which not only have anti-inflammatory properties but also initiate cell differentiation and regulate wound healing as well as the regeneration process [9,10]. Due to their good composition, they are dedicated mainly to the treatment of acute DE. Moreover, there is strong evidence that blood-derived products are superior and more effective than artificial tears in SSDE [11,12] due to the fact that their composition is similar to natural tears and autologous preparation.

There are numerous preparation methods for blood-derived products. We can obtain AS, PRP, platelets rich in growth factors (PRGF), and platelet lysate.

Autologous serum was first introduced in 1975 to treat ocular surface disease [13]. Later, Fox et al., used the serum to treat dry eyes linked to the SS in 1984 [14]. The serum is obtained from the blood collected without an anticoagulant by a physiological clotting process.

PRP is an autologous blood product with platelet concentrations above baseline [15]. The first description of PRP came from haematology, where it was used as a transfusion product to treat thrombocytopenia in the 1970s. Ref. [16] PRP, due to its huge concentration of platelets suspended in a small volume, has versatile immunomodulatory and anti-inflammatory active components. PRP plays a vital role in regeneration, cell proliferation, tissue remodeling, and repair, as well as the angiogenesis process.

The role of AS and PRP in ocular surface damage has been an area of interest for numerous ophthalmologists. The purpose of this study was to determine the differences in the composition of 100% AS and 100% PRP eye drops and to determine the impact of growth factor levels on clinical outcomes in the treatment of severe DE in pSS.

## 2. Materials and Methods

This is an interventional, non-randomized, comparative study with patients recruited from the ophthalmology clinic of the Medical University of Lublin, Poland. The research was approved by the Local Ethics Committee of the Medical University of Lublin (no. KE-0254/61) and followed the tenets of the Declaration of Helsinki.

### 2.1. Participants

Twenty-two female patients with a diagnosis of pSS in accordance with the 2002 American-European Consensus Group Criteria for Sjögren’s Syndrome (AECG) [17] took part in the study. All the patients who met the criteria and agreed to participate were included. All participants were volunteers. They received a full explanation of the research aims and methods, and then informed consent was obtained from all of them. Data and blood samples were coded with the study number.

Inclusion criteria:

1. Women with dry eye disease due to primary Sjogren syndrome diagnosed according to the AECG 2002 classification [17];
2. age ≥ 18 years old;
3. no general disease;
4. the same diet habits (a balanced diet according to the patient’s opinion);
5. Ocular Surface Disease Index questionnaire (OSDI ≥ 23), at least moderate DE symptoms;
6. tear break-up time (TBUT < 10 s);
7. Schirmer’s test (≤10 mm);
8. abnormal ocular surface staining—Oxford Scale ≥ 2.

Exclusion criteria:

1. pregnancy or breastfeeding;
2. other connective tissue disorders;
3. diabetes;
4. acute or chronic general infection, infectious disease;
5. cancer;
6. blood pathology or blood-transmitted disease;
7. chronic liver or kidney disease;
8. anticoagulant or antiplatelet therapy;
9. use of haemoderivates within 3 months before the enrolment;
10. glaucoma;
11. previous history of ocular trauma, ocular surgery within 6 months;
12. use of contact lenses;
13. punctal plug.

Previously topically used medications (drops) were stopped 48 h before starting the AS or PRP therapy. A comprehensive ophthalmological examination, medical history, demographic data, and ocular evaluation were all recorded.

### 2.2. Clinical Examination

The patients who met the inclusion criteria and consented to participate in the study were divided into 2 groups and advised to use the derivatives (AS, PRP) 1 drop 5 times per day. Participants were assessed by a masked research ophthalmologist (not participating in the therapeutic process) at baseline, 1 month, and 3 months into the treatment. The OSDI questionnaire was filled out. The clinical examination was performed by a trained masked ophthalmologist and included a visual acuity measurement and a comprehensive anterior segment evaluation with slit lamp biomicroscopy. Tests of tear function, such as the Schirmer test and tear breakup time, were performed, as well as an evaluation of the cornea and conjunctiva surfaces with ocular surface staining in agreement with the Oxford scale, conjunctival hyperaemia, and the Meibomian gland parameters. The tests were performed as described below before the treatment and at a 1-month and 3-month follow-up after the treatment.

#### 2.2.1. Ocular Surface Disease Index Score (OSDI)

The OSDI standard self-assessment questionnaire consisted of 12 validated questions divided into 3 parts devoted to visual-related function, ocular symptoms, and environment-triggered symptoms [18]. Each question was graded with a score ranging from 0 to 4. The total score was the sum of the scores for all questions answered multiplied by 25 and divided by the number of questions answered. According to the OSDI score, the severity of DE was classified as normal ocular surface (0–12 points), mild DE (13–22 points), moderate DE (23–32 points), and severe DE (33–100 points).

#### 2.2.2. Best Corrected Visual Acuity (BCVA)

Snellen VA testing was performed using commercial Snellen projectors according to a routine, standard-of-care examination, and a score was given on the basis of a line assignment method using the last line where at least 3 of 5 letters were correctly identified. Then, VA was converted to a logMAR scale according to the pattern: logMAR = −1 * log10 (snellen_frac).

#### 2.2.3. Schirmer Test I without Anaesthesia

A Schirmer strip (Whatman filter paper number 41) was placed in the lateral third of the inferior conjunctival fornix. Patients were asked to close their eyes. The amount of wetting on the filter paper was recorded after 5 min.

#### 2.2.4. Oral Schirmer Test

Moreover, unstimulated salivary Schirmer tests were performed in the morning to measure unstimulated salivary flow. Patients were not allowed to eat, drink, smoke, or brush their teeth for 2 h before the examination. Participants were asked to sit upright in a chair, swallow all the saliva in the mouth before the test, and not swallow during the test. Moreover, patients were requested to open their mouths and rest their tongues on the hard palate so as to prevent the inadvertent wetting of the test strips. The results were recorded after 5 min [19]. The test was performed to confirm the oral dryness that accompanies SS.

#### 2.2.5. Tear Break-Up Time (TBUT)

A fluorescein strip with alcaine (Alcaine, Alcon) was placed at the inferior conjunctival fornix without touching the cornea. Patients were instructed to blink a few times (3–5) and to abstain from blinking during the examination. The measurements were taken during a slit lamp examination with cobalt blue light. The time taken for the first dry spot to appear after blinking was recorded, then three measurements were taken, and the average was calculated and used for analysis.

#### 2.2.6. Ocular Surface Fluorescein Staining—Oxford Scale

The Oxford scale for corneal and conjunctival fluorescein staining patterns was graded from zero to five according to previously established criteria [20].

#### 2.2.7. Conjunctival Hyperaemia

The level of bulbar hyperaemia was evaluated using the Brien Holden Vision Institute scales, which included a 5-point bulbar redness photographic scale where 0 meant no hyperaemia, 1 = very slight, 2 = slight, 3 = moderate, and 4 = severe [21].

#### 2.2.8. Meibomian Gland Parameters

The quality of meibomian gland secretion was assessed during a slit lamp examination in each of the eight glands of the central third of the lower lid on a scale of 0 to 3 for each gland: 0—clear, 1—cloudy, 2—cloudy with debris (granular), and 3—thick like toothpaste (total score range 0–24).

Expressibility was determined on a scale of 0 to 3 in five glands in the lower or upper lid, according to the number of glands expressible: 0—all glands, 1—three to four glands, 2—one to two glands, and 3—no glands [22].

#### 2.2.9. Adverse Events

Patients were asked about any adverse effects of the topical treatment at the 1-month and 3-month follow-up visits.

### 2.3. Autologous Serum and Platelet-Rich Plasma Preparation

AS and PRP preparation were performed under strict sterility conditions using a laminar flow hood on all patients. Patients fasted before blood sampling, which took place in the morning between 7 and 9 o’clock. Initially, 5 mL of peripheral whole blood was collected by venipuncture in the arm using EDTA tubes (EDTA/4.9 mL, Sarsted Monovette, Sarstedt UK, Leicester, UK) to evaluate the number of platelets (PLT) and white and red blood cells.

#### 2.3.1. AS Preparation

The blood was collected into 5 mL vacuum probes without anticoagulant or clot activator (Serum/4.9 mL, Sarsted Monovette). The samples were left standing in an upright position for 1 h at room temperature (22–26 °C) to ensure blood clot formation. The tubes were centrifuged for 10 min at 3000 rpm to allow the separation of serum from the clotted blood. Then the supernatant serum was transferred into new tubes and centrifuged for 10 min at 3000 rpm. The serum was introduced into a Millex filtration unit (0.45 um, Durapore, Millipore, Madrid, Spain) and dispensed without dilution into 2 mL Eppendorf tubes for further analysis and as the final product to be administered by the participants in the form of eye drops.

#### 2.3.2. PRP Preparation

Peripheral blood was collected in 9-mL vacuum tubes containing 1 mL of 3.8% sodium citrate as an anticoagulant (Monovette, Sarsted). PRP was prepared in a single-step technique and centrifuged at 1400 rpm for 10 min according to the protocol previously described by Alió. This resulted in the formation of three layers: mostly red blood cells at the bottom, a thin middle buffer layer of leukocytes, and platelet plasma at the top of the tube. Aggregation or activation of platelets were not induced. All PRP top layers were collected, avoiding the buffy coat, and divided into smaller portions (2 mL Eppendorf tubes) for further analysis and as the final product to be administered by the participants as eye drops.

Manipulation of the blood, autologous serum, and platelet-rich plasma was performed under strict sterility conditions inside the laminar flow cabin with sterile disposable materials. The blood was collected from all patients on the same day. Moreover, autologous serum and platelet-rich plasma were prepared immediately and divided into 2 mL Eppendorf tubes for further analysis. The time between blood collection, preparation of blood products, and their transfer to the laboratory did not exceed 4 h. During that time, the samples were stored at 4 °C. The transport to the laboratory was carried out in a transport refrigerator that maintains a temperature of 4 °C. Morphology determinations and ELISA tests were performed immediately after the samples delivery to the laboratory. The final blood products, AS and PRP, were transferred into opaque eye drop bottles to protect them from ultraviolet light and labeled with a name and a date. Each patient received 12 bottles for the whole treatment period and a leaflet with instructions on how to apply and store the product. The currently used bottle should have been stored at 4 °C for 7 days (one bottle for one week) and the remaining bottles at −20 °C in a fridge until the day of use.

### 2.4. Haematological Analysis

The evaluation of the total amount of blood cells (red cells, leukocytes, and platelets) in whole blood, AS, and PRP was performed twice for each sample using automated haemocytometry (Analizator Sysmex XN 2000, Sysmex, Kobe, Japan). The means of the two measurements were used for further calculations.

### 2.5. Quantification of Growth Factors, Fibronectin, and Substance P

A commercially available enzyme-linked immunosorbent assay (ELISA) kit (R&D System Minneapolis, Minneapolis, MN, USA) specific for GFs and protein levels such as fibroblast growth factor (FGF), epidermal growth factor (EGF), vascular endothelial growth factor (VEGF), platelet-derived growth factor (PDGF), nerve growth factor (NGF), transforming growth factor (TGF-b), insulin-like growth factor (IGF), fibronectin, and substance *p* were used to quantify their amount in AS and PRP on the day of blood collection and haemoderivate preparations. The molecules were chosen according to their functions in the corneal regeneration process. The procedures were carried out following the manufacturer’s instructions on the attached datasheet for the EuroImmune Analyzer. All samples were measured twice, and the means of the two measurements were taken into consideration in further investigations. Results were expressed as pg/mL.

**Figure 1:**
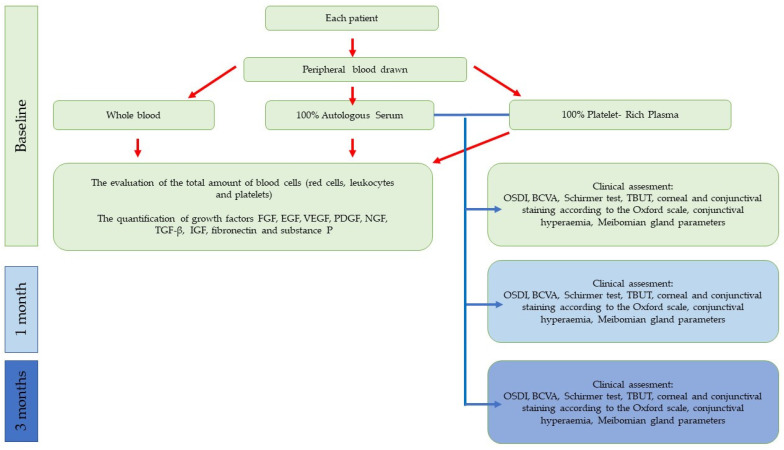
Asummary of the design of the study.

A summary of the design of the study is presented in Figure 1.

### 2.6. Statistical Analysis

#### 2.6.1. Sample Size Calculation

The statistical power was assumed at 80%, alpha < 0.05, and a relative difference of at least 30% when calculating the sample size for the study. The investigators were required to test at least 44 samples in the autologous serum arm vs. 44 in the platelet-rich plasma arm. The authors adhered to the adopted study plan and the aforementioned sample sizes. Each patient provided two test tubes, i.e., one containing AS and the other containing PRP. Subsequently, each sample vial was subjected to laboratory measurements twice.

#### 2.6.2. Statistical Analysis

Categorical variables were described using integer numbers and percentages. Numerical traits were depicted through their mean, standard deviation, median, lower-to-upper quartile, and minimum-to-maximum values.

The normality of the distribution was assessed using the Shapiro-Wilk W test. The homogeneity of variances was tested using Levene’s test.

The statistical significance of differences at baseline was assessed using generalized linear models due to non-normal and left-skewed distributions. Generalized linear models with repeated measures were fitted in order to estimate the dynamics of both continuous and discrete variables throughout the observation period, incorporating a set of explaining variables and being controlled for the patients’ age.

A level of *p* < 0.05 was considered statistically significant. All the statistical procedures were carried out using STATISTICA™, release 14 (TIBCO Software Inc., Palo Alto, CA, USA).

## 3. Results

### 3.1. Patients

Twenty-two females were included in the study and divided into two equal groups, the first treated with AS and the second with PRP. All participants completed the study, with none dropping out. The mean age in the PRP group was 62.18 ± 8.27 years, and in the AS group it was 62.86 ± 10.89 years, *p* > 0.05. There were no significant differences between the groups at baseline in terms of OSDI score, the Schirmer test, TBUT, the Oxford grade scale, conjunctival hyperaemia, or Meibomian gland parameters. Thus, the studied groups were all clinically well-matched (Table 1 and Table 2).

### 3.2. Growth Factor Level

There was almost no detectable level of red and white blood cells in AS and PRP, which indicated that the preparation was highly purified. The mean number of platelets in whole blood was 194.23 ± 63.12 × 10^3^/uL, and in PRP, it was 297.058 ± 100.63 × 10^3^/uL, *p* < 0.05. The procedure described above resulted in a mean 1.5-fold increase in platelet concentration.

The concentration of selected GFs in blood-derived eye drops (PRP vs. AS) was analyzed, and the results are presented in Table 3. Significant differences were observed in the concentration of FGF (4.42 ± 0.86 vs. 15.96 ± 7.63, *p* < 0.0001), EGF (4.98 ± 0.97 vs. 39.06 ± 20.18, *p* < 0.0001), fibronectin (929.6 ± 111.5 vs. 823.64 ± 98.49, *p* = 0.0005), VEGF (175.45 ± 65.93 vs. 717.35 ± 488.15, *p* < 0.0001), PDGF AB (619.6 ± 117.30 vs. 349.66 ± 79.82, *p* < 0.0001), NGF (85.22 ± 23.49 vs. 8.29 ± 9.06, *p* < 0.0001), PDGF (935.38 ± 434.26 vs. 126.66 ± 54.41, *p* < 0.0001), and substance P (112.58 ± 27.28 vs. 127.51 ± 26.56, *p* = 0.0125) in PRP compared to AS. The level of TGF-β was undoubtedly higher in AS than in PRP (1031.37 ± 330.23 vs. 726.03 ± 298.95, *p* = 0.0004). No significant differences between AS and PRP were observed in the IGF concentration (Table 3).

### 3.3. Clinical Status

#### 3.3.1. OSDI

The baseline mean OSDI score fell from 79.85 ± 13.12 to 67.07 ± 11.57 (*p* < 0.0001) in the AS group, whereas the mean value changed from 79 ± 12.71 to 59.81 ± 10.31 at the 3-month visit in the PRP group (*p* < 0.0001). A reduction in the OSDI questionnaire was noticed in 100% of patients in the PRP group and in 77.3% of the AS group as compared to baseline (*p* = 0.0031). There was a significant difference in the mean change of the OSDI score between and within the groups (*p* = 0.0031, *p* < 0.0001). In both groups, patients reported significant improvement in their symptoms after the treatment. The results are shown in Table 4.

#### 3.3.2. BCVA

The mean BCVA (logMAR) of all participants improved in the AS group from 0.06 ± 0.07 to 0.04 ± 0.04, *p* = 0.001, and in the PRP group from 0.21 ± 0.23 at baseline to 0.09 ± 0.12 after the treatment, *p* = 0.0046, respectively. Improvement of BCVA was observed in 45.5% of patients in the PRP group and 13.6% in the AS group (*p* = 0.0063) after 3 months of therapy.

A statistically significant difference was also observed in the improvement of BCVA during the treatment period between both groups *(p* = 0.0063) (Table 4).

#### 3.3.3. Schirmer Test

There was no difference in the baseline mean value of the Schirmer test in the studied groups. In PRP, it was 2.95 ± 2.8 mm, and in AS, 2.91 ± 2.79. However, after 3 months, it improved to 5 ± 2.86 and 4 ± 3.41, respectively. There was a significant difference between the values within the groups after the treatment. Interestingly, there was no significant difference between the two groups after 3 months of therapy (Table 5).

Oral Schrimer test (mm).

No significant changes in the mean value of the oral Schirmer test were detected in the researched groups.

#### 3.3.4. TBUT

The TBUT scores increased in the AS group from the baseline of 4.72 ± 1.8 s to 5 ± 2.7 s at the last follow-up visit, while in the PRP group, the baseline TBUT was 5.36 ± 1.8 and went up to 5.95 ± 2.3 after 3 months. A significant improvement was observed in each group, *p* < 0.0001, but not between the groups after the treatment, *p* = 0.1714 (Table 5).

#### 3.3.5. Ocular Surface Staining

At baseline, there were 40.9% of patients with grade 4 fluorescein staining, 27.3% with grade 3, and 31.8% with grade 2 in the AS group, whereas in the PRP group, there were 45.4%, 27.3%, and 27.3%, respectively. After the 3-month treatment, there were in the AS group 31.8% of patients with grade 4, 31.8% with grade 3, 27.3% with grade 2, and 9.1% with grade 1; in the PRP group, 13.6%, 40.9%, and 45.5%. A reduction in Oxford staining score values was observed in each group; however, there was no statistically significant difference between the groups after the treatment. The results during and after the treatment are presented in Table 6.

#### 3.3.6. Conjunctival Hyperaemia

Conjunctival hyperaemia decreased significantly in both groups after the treatment. Nevertheless, there was no statistically significant difference between the groups (*p* = 0.1089) (Table 6).

The oral Schirmer test and tear film meniscus did not change significantly.

#### 3.3.7. Meibomian Gland Parameters

There were no significant changes observed in meibomian gland parameters during and after the treatment between and within the groups (Table 7).

#### 3.3.8. Safety

Participants did not report any discomfort or irritation during the treatment period. No temporary or permanent adverse events were noticed during or after the biological product therapy.

### 3.4. Relationship between GFs and Clinical Outcomes

In the AS, there were correlations between TGF-β and EGF (r = 0.68, *p* = 0.013), TGF-β and PDGF-AB (r = 0.81, *p* = 0.001), and PDGF and PDGF-AB (r = 0.79, *p* = 0.001). There was a significant positive correlation between TGF-β and EGF (r = 0.65, *p* = 0.0039), PDGF (r = 0.09, *p* = 0.7221), PDGF AB (r = 0.44, *p* = 0.0709), and VEGF (r = 0.56, *p* = 0.0178) in PRP. In addition, remarkable interactions were found between EGF and FGF (r = 0.27, *p* = 0.2155), VEGF (r = 0.58, *p* = 0.0039), PDGF AB (r = 0.69, *p* = 0.0002), and NGF (r = 0.45, *p* = 0.0316) in PRP. Moreover, a positive correlation was observed between NGF and EGF (r = 0.45, *p* = 0.0316), FGF (r = 0.39, *p* = 0.039), PDGF, and PDGF-AB (r = 0.764957265, *p* = 0.0001) in PRP.

There was also a positive correlation between the PLT number and the PDGF level (r = 0.9, *p* = 0.037) in PRP. However, no correlation was noted between PLT and other GFs.

The relationship between the concentration of the GFs and the mean change of the clinical outcomes was analyzed. There was an interaction between the reduction of OSDI score and the level of NGF in both groups (AS r = 0.82, *p* < 0.0001 vs. PRP r = 0.7, *p* < 0.0001), whereas the statistically significant correlation between the mean OSDI change and the level of VEGF (r = 0.6, *p* = 0.0012), PDGF (r = −0.64, *p* = 0.045), and substance *p* (r = 0.56, *p* = 0.0324) was observed only in the PRP group. In the AS group, there was a negative relationship between the OSDI score and the level of TGFβ (r = −0.19, *p* = 0.017) and EGF (r = −0.48, *p* = 0.009).

Interestingly, the improvement of BCVA was significantly correlated with the concentration of PDGF-AB (r = −0.22, *p* = 0.0237), PDGF (r = −0.19, *p* = 0.006), and substance *p* (r = 0.7, *p* = 0.0111), but only in the PRP group.

Regarding the Schirmer test, a correlation was noticed between the levels of FGF (r = −0.664, *p* = 0.001), EGF (r = −0.48, *p* = 0.0034), and TGFβ (r = −0.32, *p* = 0.0452) in the AS group and only with NGF in the PRP group (r = 0.57, *p* = 0.0013).

The mean increase of the TBUT value was correlated with TGFβ (r = −0.48, *p* = 0.023), IGF (r = −0.47, *p* = 0.027) in the AS group, while in the PRP group it was correlated with IGF (r = −0.3, *p* = 0.0457) and TGFβ (r = −0.68, *p* < 0.0146).

The mean reduction of Oxford ocular surface staining was connected with the FGF level (r = −0.58, *p* = 0.0025) in the AS group and in the PRP group with PGDF (r = −0.35, *p* = 0.048), PGDF-AB (r = −0.15, *p* = 0.0457), and NGF (r = 0.47, *p* = 0.045).

Regarding the decrease of conjunctival hyperaemia, there was a strong statistical interaction with FGF (r = −0.64, *p* = 0.014), PDGF (r = −0.48, *p* = 0.013), and PDGF-AB (r = −0.52, *p* = 0.0366) in the AS group, and with FGF (r = −0.57, *p* = 0.001), PDGF (r = −0.5, *p* = 0.0258), and PDGF-AB (r = −0.46, *p* = 0.0212) in the PRP group.

Interestingly, the level of substance *p* (r = 0.51, *p* = 0.018, and r = 0.52, *p* = 0.02) and EGF (r = −0.33, *p* = 0.0091 and r = −0.22, *p* = 0.0082) had an impact on the quality and expressibility of meibomian glands only in the PRP group.

## 4. Discussion

Haemoderivates appear to be a promising strategy for DE; however, there seems to be no consensus regarding AS and PRP preparation, concentration, or frequency of application. Some authors believe that the higher the platelet count and the higher the number of growth factors inside, the better the treatment results. Thus, the therapeutic benefit of blood-derived products might be explained by their composition, which is very much like natural human tears.

Our study is one of the first to compare GF concentrations in 100% AS and 100% PRP and attempt to determine their influence on clinical results after the treatment of DE in pSS patients. So far, no consensus has been achieved regarding the preparation of blood derivatives; thus, products with different compositions and clinical effects are obtained. It was demonstrated that TGF-β concentration was 5 times higher in the AS than in tears; therefore, some authors suggested diluting it to diminish the concentration of these anti-proliferable and pro-fibrotic factors [12,23]. However, the dilution process led to a decrease not only in TGF-β concentrations but also in other beneficial GFs with regenerative potential properties. No data is currently available on what concentration of AS or PRP is most appropriate for the treatment of ocular surface disease. Many studies have demonstrated the efficacy and safety of undiluted concentrations of blood derivative eye drops in the corneal healing process by providing higher concentrations of the GFs [24]. There is no clear clinical evidence to favor any specific concentration. However, in vitro experiments on human and rabbit corneal epithelial cells proved that 100% concentration was optimal for cell migration and the corneal reepitalization process [25,26,27,28,29]. A 100% concentration of the products was chosen to avoid any potential contamination during the dilution process. Moreover, it is widely known that a preservative-free preparation reduces the risk of the chemical toxicity of the diluents on the damaged ocular surface. In our opinion, the inconvenience for patients with 100% concentrations of blood derivative eye drops might be the large volume of the blood collection and the likelihood of repeated blood draws. Bearing in mind the various methods and commercially available devices used to obtain these blood products, the abovementioned preparation protocol was chosen. In our opinion, they are easy and simple to perform, inexpensive, and, most importantly, effective in our clinical practice.

In this study, a statistically significant increase in BCVA, the Schirmer test, TBUT, meibomian gland parameters, and a reduction in the OSDI scores, Oxford staining, and conjunctival hyperaemia were all observed in each group, but within the groups, the mean changes in BVCA and OSDI scores were more significant in the PRP group. It is worth emphasizing that the patients in the PRP group presented worse BCVA at baseline compared to the AS group, which was not intended and constituted a certain limitation of the study. Thus, the expected improvement in visual acuity may be greater in the PRP group than in the less-affected AS group. That fact was not enough to draw the conclusion that PRP had a statistically significantly better effect on improving visual acuity than AS. The improvement of VA and alleviation of subjective symptoms are the most expected and desired effects of the treatment in this chronic disease, which significantly improves the patient’s quality of life. A reduction in the OSDI questionnaire in this study was noticed in 100% of patients in the PRP group and 77.3% in the AS group compared to baseline (*p* = 0.0031). Improvement of BCVA was observed in 45.5% of patients in the PRP group and 13.6% in the AS group (*p* = 0.0063) after 3 months of therapy. The effect on visual acuity could be secondary to the smoothening of ocular surface irregularities or improving subjective symptoms of DE.

Interestingly, according to the statistical analysis, the mean change of TBUT (less than 1 s in both groups) after the treatment was significant in both groups. We do not know exactly how this translates into clinical practice since many factors are responsible for the improvement of the clinical condition and symptoms at the same time, and sometimes a small change in one of them significantly affects the whole condition. Moreover, some relationships between the TBUT improvement and growth factors were also noted, which would be discussed afterwards.

However, some of the results achieved by other authors are worth citing. According to the meta-analysis performed by Jongkhajornpong, PRP significantly reduced OSDI score and ocular surface fluorescein staining and increased TBUT more than AS [30]. The findings of this study were also consistent with previous systemic reviews on SS, which showed short-term benefits of undiluted AS in alleviating symptoms and reducing corneal staining compared to other biological tear substitutes. In agreement with the research made by Cho, a reduction in OSDI and the Oxford scale [28] was also observed.

Nevertheless, comparing different studies on the subject of PRP and AS was challenging because of the differences in the preparation protocols of the blood products and the different outcome assessments across the studies.

The Cochrane meta-analysis of randomized controlled trial reports devoted to the efficacy of 20% AS in comparison to artificial tears suggested that 20% AS eye drops alleviate DE symptoms better than artificial tears, but only for the first period of treatment (2 weeks). The data for the long-term efficacy period was ambiguous [12]. Interestingly, researchers Urzua, Kojima, and Celebi observed a better reduction of OSDI values, improvement in the Schirmer test, elongation of the TBUT, and the Oxford scale in patients treated with AS versus artificial tears; however, Cochrane investigators did not consider them clinically significant when preparing their report [31,32,33]. On the other hand, there was no difference in the Schirmer test or the ocular surface staining score between those two groups in a study by Wang [34]. Surprisingly, few studies failed to demonstrate significant improvement in objective clinical outcomes such as TBUT, the Schirmer test, or ocular staining [35], whereas some of them revealed controversial results. Such a discrepancy might result from a different study population and may be due to circulating antibodies and pro-inflammatory cytokines present in severe dry eye [31,33,36].

Concerning the application of PRP for the management of dry eye, a successful result with minimal complications has been reported by Alio et al., who observed an improvement of symptoms in 87.5% of participants and decreased corneal fluorescein staining (CFS) in 76.1% of cases. Moreover, 28.8% of patients improved by at least one line of BCVA, and they observed a significant decrease in OSDI scores [37]. Similarly, Garcia-Conca et al., conducted a study with hyposecretory dry eye patients treated with PRP and sodium hyaluronate artificial tears for 30 days (6 times per day), obtaining statistically significant improvement in the PRP group in BCVA and the Schirmer test values and a reduction in OSDI score, ocular surface staining, hyperaemia, and osmolarity when compared to artificial tears [38].

Additionally, Rawat reported an OSDI score reduction (*p* < 0.001) and a significantly higher improvement in TBUT and CFS scores (*p* < 0.05) after 3 months of treatment with PRP compared to artificial tears. There was no significant difference observed in the post-treatment Schirmer test score between the two groups (*p* = 0.44) [39]. Meibomian gland parameters were also assessed, but without any significant changes in the tear film quality or quantity after the treatment in either group. In contrast to our results, Fahmeeda Murtaza reported an improvement in evaporative DE after PRP therapy [40]. This difference in the results may be due to the patients’ baseline clinical condition. In our study, the patients had severe dry eyes according to the OSDI, but in Murtaza’s research, they had mild or moderate dry eyes according to the Canadian Dry Eye Assessment questionnaire. Interestingly, our patients used only PRP or AS 5 times a day as monotherapy, but the Canadian study participants were allowed to continue baseline topical medications or previous therapy (artificial tears, cyclosporine, steroids, lifitegrast, oral Omega-3 supplements, hot compresses, and lid wipes) together with PRP drops used 6 times a day, thus the achieved effects may not only be due to PRP. What is more, the PRP eye drops production protocols were different. In our study, the tubes with 3.8% sodium citrate and one-step centrifugation methods were used, whereas Murtaza worked with the tubes with 2 mL of acid-citrate-dextrose solution A and 2-step centrifugation. The Canadian authors did not show the final PLT concentration or the concentration of the GFs in PRP eye drops. As far as the clinical improvement of the tear film quality and quantity was concerned, they measured non-invasive break-up time by a Keratograph 5 M, tear meniscus height, etc., whereas we performed invasive BUT. We believe that the differences in the qualifications of patients, diagnosis, and treatment, as well as the preparation of the blood product presented above, were responsible for obtaining completely different effects from the PRP therapy.

Some authors performed studies concerning PRP injection to the lacrimal gland of SSp, obtaining relief from the signs and symptoms of DE [41,42].

In spite of these encouraging results, there is limited availability of clinical trials proving the superiority of PRP treatment in dry eye disease in SS over autologous serum therapy. Blood derivatives contain bioactive ingredients that are missing in artificial tears. It is believed that PRP provides more advantages than AS eye drops as PRP has a more beneficial composition and its regenerative effect is based on a different concentration of several growth factors. Most of them influence corneal epithelial healing. According to some authors, platelet-derived eye drops provide larger amounts of NGF, PDGF, and fibronectin than AS [43,44]. Platelet-rich plasma, due to its huge concentration of platelets suspended in a small volume, has versatile immunomodulatory and anti-inflammatory active components. They play a vital role in regeneration, cell proliferation, tissue remodeling, and repair, as well as the process of angiogenesis.

However, there is still a lack of comparative studies confirming this. According to the Dry Eye WorkShop (DEWS) II report [45], blood products constitute one of the most powerful therapeutic strategies in dry eye disease but are recommended as step 3 when the previous treatment has proven inefficient [46].

In our study, significant differences in the concentration of growth factors between PRP and AS were noted. The level of TGF-β was undoubtedly higher in AS than in PRP. No significant differences between AS and PRP were observed in the concentration of IGF. The observed dependences indicate a complex mechanism of action and synergy in the cooperation of the growth factors with each other. Those data may support the hypothesis that a higher level of growth factors may better relieve the symptoms and enhance corneal epithelial healing. All of this may lead to a reduction in OSDI and ocular surface fluorescein staining. For this reason, PRP has superior efficacy over AS.

Our study revealed that there was a higher FGF level in AS correlating with the improvement in the Schirmer test, Oxford staining, and the reduction of conjunctival hyperaemia in both groups, which was in agreement with Xiong research confirming that FGF alleviates DE by modulating the inflammation response and promoting the healing process [47].

Lacrimal glands increase the expression of EGF after corneal epithelium injuries via the trigeminal nerve. Therefore, EGF plays an important role in the healing process by controlling/promoting epithelial cell proliferation and migration and preventing apoptosis. Moreover, EGF increases the number of goblet cells and muc−1 expression, thus contributing to tear film stability [48] by the mucin layer. In our study, we observed a higher level of EGF correlating with an improvement in the Schirmer test and a reduction in OSDI score in the AS group. Xiao, in an animal model of dry eye, also noticed a positive correlation between EGF, longer TBUT, and lower ocular surface staining. Moreover, Alio noticed an increase in conjunctival goblet cells after 1 month of PRP treatment. The abovementioned observations were discordant with the research published by Ripa et al., They noticed a significantly lower EGF level and a lower reduction in OSDI in patients with a systemic autoimmune disease [49].

Ohashi et al., found a correlation between EGF in tears and the Schirmer test—a decreased level of EGF in tears was observed in SSp. It is widely known that the lacrimal gland’s ability to produce tears is decreased in SS, so the quantity and quality of tears are extremely important to preserve the biological activity of the tear proteins and protect the ocular surface. When the lacrimal gland does not work properly, we can deliver GFs such as EGF to preserve ocular surface function.

Despite very advanced dry eye and a higher level of VEGF in AS compared to PRP, we did not observe neovascularization in our patients. This may be due to the inhibitory effect of the other GFs or the balance between the proangiogenetic and antiangiogenetic factors. The concentration of VEGF had a statistically significant correspondence with the reduction in OSDI values in the PRP group and, what is more interesting, with the improvement of the Schirmer test in the AS group. There is still no information on how exactly this GF can influence the abovementioned outcomes. However, some animal studies reported that VEGF was detected in the lacrimal glands of rat models [50]. VEGF can promote proliferation, differentiation, and migration of vascular endothelial cells and fibroblasts, accompanied by upregulation of TGFβ miRNa, as well as participate in the pathological process of inflammation. Thus, this factor may be both a reason for and an effect of chronic inflammation and aggravate the DE. This relationship remains to be investigated. Trophic growth factors have a crucial role in corneal epithelium integrity and the reepithelialisation process. Unfortunately, the exact mechanism has not been fully explained.

Normally, corneal epithelial cells are permanently renewed by mitosis; corneal neurotrophic factors play a fundamental role in maintaining cornea health. Generally, NGF has a decisive function in the regulation of growth, maintenance, proliferation, differentiation, and survival of corneal nerve and sensory neurons, corneal sensitivity, and even tear production. Moreover, it may be potentially responsible for modulating the proliferation of corneal epithelial cells.

It is assumed that the ocular surface and the lacrimal gland are connected by nerves, and their impairment might evoke DE. Deterioration in corneal sensitivity induces a decrease in tear production and suppresses the blinking reflex, resulting in a hyperosmolar environment and inducing inflammation. All of this reduces the expression of epitheliotrophic neuromediators/GFs.

Cortes et al., measured the NFG level in tears in patients with moderate dry eye syndrome. They observed a negative correlation between NGF and OSDI results [51]. Similar results were obtained in our study. In PRP eye drops, there was a statistically significant higher level of NGF compared to AS eye drops. In this study, a higher level of NGF corresponded to a bigger decrease in OSDI value after the treatment (*p* = 0.0016) was noticed. Moreover, a correlation between the level of NGF and the improvement of the Schirmer test value and Oxford staining score in the PRP group was observed. This observation might be explained by the pleiotropic effect of NGF on the ocular surface. NGF is known not only to influence and modulate corneal sensitivity and corneal and conjunctival epithelial proliferation and differentiation, but it also controls immune reactions and stimulates mucin production by goblet cells [52,53]. Likewise, lacrimal gland production increased with higher levels of NGF [51,54]. This is due to the presence of nerve endings in the cornea and the neural reflex at the afferent nerve endings. Some authors observed higher nerve growth factor (NGF) levels in tears in dry eye patients compared to healthy participants. Moreover, they found a direct correlation between NGF tear levels and the severity of dry eye (*p* = 0.009) [51]. Nonetheless, the increase in NGF tear levels may be the consequence of an increased release by the lacrimal gland, which is NGF-dependent.

The NGF level was definitely higher in PRP than in AS (85.22 ± 23.49 vs. 8.29 ± 9.06, *p* < 0.0001). This growth factor is responsible for cornea health and sensitivity improvement. Therefore, it helps to improve not only local conditions but also subjective symptoms. Nowadays, recombinant human NGF (rhNGF) as an eye drop solution is approved for the treatment of neurotrophic keratopathy. Observational studies results are encouraging; however, due to their high cost, their use is limited [55]. In severe dry eye, disturbed corneal sensation together with decreased tear production cause damage to the ocular surface, which can be prevented by NGF application aimed at improving corneal innervation and tear secretion. Bearing in mind the price of rhNGF and its poor concentration in AS, PRP proves to be superior.

According to the literature, substance P plays a crucial role in the pathogenesis of dry eye and autoimmune disorders such as SS [56], influencing the maturation of antigen-presenting cells at the ocular surface after stress exposure. Moreover, substance *p* is one of the main neuropeptides of corneal innervation and a pain modulator. It protects corneal epithelial cells from hyperosmotic stress-induced apoptosis [57].

According to experts, substance P establishes synergism with other GFs such as EGF, IGF, and FGF, influencing epithelial cell growth, migration, and adhesion.

Sang et al., described an increased expression level of substance P in the cornea and conjunctiva that was correlated with the increased CFS scores (*p* < 0.001) [58]. In our study, we did not observe such a dependency. The level of substance P was statistically significant between the two groups, with a slightly higher level in the AS group; however, we achieved a positive correlation between mean changes in OSDI and BCVA and meibomian gland parameters in the PRP group. Unfortunately, there is still little known about the contribution of substance P to DE.

PDGF is a modulator of chemotaxis and the cellular mitosis of fibroblasts. Moreover, it stimulates the expression of other factors, especially TGFβ. In PRP, we observed its higher concentration due to platelet degranulation. Interestingly, a negative correlation between the level of PDGF and PDGF-AB and the mean change of BCVA, OSDI score, Oxford staining, and hyperaemia [59] was found in our study.

It is widely known that TGF-β is a multifactorial GF and has antiproliferative properties in a dose-dependent manner. It was demonstrated that TGFβ may suppress corneal wound healing and promote corneal stromal fibrosis—the disorganized extracellular matrix generated by myofibroblasts is responsible for corneal opacities. Therefore, a lower concentration is more desirable in the healing process. A lower level of TGFβ in PRP stimulated corneal regeneration due to weak conversion of stromal fibroblasts to myofibroblasts and scar formation [60]. TGF-β was observed in corneal epithelial cells, stroma, and endothelium, and it seems to act synergistically with PDGF, EGF, and fibronectin in the tissue repair process. In our study, a statistically significant correlation between the level of TGF-β and the reduction of conjunctival hyperaemia was proved in both groups. Its level in AS is approximately 5 times higher than in tears; thus, according to some authors, AS should be diluted to prevent its potentially negative effects [27]. However, dilution reduces the concentration of other factors. According to the literature, undiluted AS was more effective than 20% AS in the epithelial cell migration and healing process, presumably due to a higher concentration of fibronectin [23,61]. For this reason, we decided to evaluate the therapeutic effects of topical 100% AS eye drops.

A statistically significant correlation between many GFs (TGFβ and EGF, PDGF, PDGF-AB, and fibronectin, as well as EGF with FGF, VEGF, PDGF, and NGF) was observed. This may indicate how complex the ocular surface regenerative process is. The healing process may be based on the interaction and synergistic mechanisms of many biologically active molecules.

It is well known that low lipid secretion and a reduction in blinking rate lead to tear film instability and tear evaporation. Ocular surface homeostasis is broken, and corneal epithelium may be injured; thus, meibomian gland function is extremely important. We noticed a negative correlation between meibomian gland parameters and the EGF level and a positive one between substance P, but only in the PRP group. Murtaza et al., reported an improvement of the TBUT and conjunctival hyperaemia in evaporative dry eye disease treated with PRP drops [40].

To understand the biology, molecular mechanism of action, and interaction between GF in PRP and AS therapy, their impact on the ocular surface in DE in SSp should be focused on further studies based on cell cultures or animal models.

The literature describes some adverse effects of hemoderivatives, such as eye irritation, discomfort, or even eye discharge. Some studies reported the potential risk of contamination of blood derivatives, but adding sterilizing filters decreased such incidences [61]. Another complication reported in the literature was limbitis in patients treated with AS for atopic keratoconjunctivitis. The problem resolved itself when the treatment was interrupted [62]. According to some authors, AS drops are composed of leukocyte degranulation products, consequently containing proinflammatory agents such as acid hydrolases and metalloproteinases, which may have a tissue destroying effect [63]. In our study, we did not observe any side effects from the hemoderivate eye drop treatment.

The storage of blood-derived products at patients’ houses was not controlled and was under the patients’ responsibility. Generally, it was reported that blood products may be used safely in both outpatient and inpatient settings, but under strict preparation and storage protocols [64,65]. The Elisa test for the level of GFs after 1 and 3 months was not performed in our study, as numerous studies suggested that blood-derived products can be stored liquid at 4 °C for up to 1 month or frozen at −20 °C for up to 3–6 months [66], but should be stored in the dark to avoid the degradation of vitamin A [12].

Lopez-Garcia reported that long-term storage of AS at 20 °C did not change the qualification level of GFs [67]. Anitua reported that PRP may be stored at −20 °C for up to 12 months without reducing the most important growth factors and proteins [68].

Interestingly, Okumura suggested that PRP eye drops stored at 4 °C for 4 weeks had a higher concentration of GFs than AS eye drops [69].

Nevertheless, evaluation of the stability of bioactive molecules should be based on cell cultures or animal models to find out their shelf life. While comparing the clinical outcomes, we found some inconsistencies, in our opinion, due to a huge number of different preparation protocols. Moreover, the quality of blood products may be affected by different preparation protocols, age, gender, race, general disease, diet, and daily routine.

Dry eye is an extremely common problem that might also display a chronic character. Over 300 million people worldwide suffer from some degree of dry eye. Blood derivates have a significant positive therapeutic role in the treatment of DE due to the huge quantity of growth factors that promote wound healing at the damaged ocular surface. The treatment with AS and PRP, as presented in our study, resulted in a significant improvement in the signs and symptoms of DE accompanying primary Sjogern’s syndrome.

Topical administration of hemoderivates appears to be a very promising strategy for DE treatment. Therefore, understanding their mechanism of action would be highly beneficial.

## 5. Conclusions

Despite the fact that the blood derivatives differ in composition, they seem to be effective and safe in the treatment of severe DE in pSS patients. The signs and symptoms of DE were reduced in both groups, but only the mean change of OSDI was statistically significant between the groups. A greater reduction in OSDI scores was observed in the PRP group. The obtained results and the composition of haemoderivates may indicate the superiority of PRP in relieving the symptoms of DE in pSS patients compared to AS.

## Figures and Tables

**Table 1:** Characteristics of the study cohort (numerical variables) (n = 22).

Analysed Trait	Platelet-Rich Plasma		Autologous Serum		*p*-Value
	M	SD	M	SD	
Age (years)	62.2	8.5	62.9	10.9	=0.8175
BCVA (logMAR)	0.21	0.23	0.06	0.07	=0.0141
OSDI score	79.0	12.7	79.8	13.1	=0.8270
Classic Schirmer test	2.9	2.8	2.9	2.8	=0.9907
Oral Schirmer test	2.1	3.0	1.1	1.3	=0.4635
TBUT (s)	5.4	1.9	4.7	1.8	=0.2566

(M—mean, SD—standard deviation).

**Table 2:** Characteristics of the study cohort (discrete variables) (n = 22).

Analysed Factor	Platelet-Rich Plasma		Autologous Serum		*p*-Value
	*n*	%	*n*	%	
Corneal fluorescein staining, Oxford scale: 2	6	27.27	7	31.82	
3	6	27.27	6	27.27	
4	10	45.45	9	40.91	=0.7222
Conjunctival fluorescein staining, Oxford scale: 2	8	36.36	6	27.27	
3	11	50	13	59.09	
4	2	9.09	3	13.64	
5	1	4.55	0	0.00	=0.8891
Conjunctival hyperaemia: 2	7	31.82	8	36.36	
3	12	54.55	10	45.45	
4	3	13.64	4	18.18	=0.9584
Quality of Meibomian gland secretion: 1	16	72.73	18	81.82	
2	4	18.18	4	18.18	
3	2	9.09	0	0.00	=0.4020
Expressibility of Meibomian glands: 1	16	72.73	16	72.73	
2	4	18.18	6	27.27	
3	2	9.09	0	0.00	=0.8544

**Table 3:** Concentration of GFs in PRP and AS.

Analyzed Factor	Study Group	Statistical Parameter					*p*-Value
		M	SD	Me	Q_1_–Q_3_	Min.–Max.	
NGF (pg/mL)	PRP	85.22	23.49	79.54	65.73–92.97	56.77–143.21	<0.0001
	AS	8.29	9.06	4.90	4.18–5.96	3.44–34.47	
PDGF (pg/mL)	PRP	935.38	434.26	749.48	633.74–1258.25	418.57–1816.98	<0.0001
	AS	126.66	54.41	105.19	95.38–171.75	50.73–229.94	
PDGF-AB (pg/mL)	PRP	619.60	117.30	615.17	547.50–694.50	404.22–878.46	<0.0001
	AS	349.66	79.82	342.98	297.23–411.95	169.30–458.00	
TGF-b (pg/mL)	PRP	726.03	298.95	777.07	630.22–867.81	91.82–1104.89	=0.0004
	AS	1031.32	330.23	943.04	787.18–1134.61	642.07–1800.80	
FGF (pg/mL)	PRP	4.42	0.86	4.30	3.82–4.57	3.51–6.14	<0.0001
	AS	15.96	7.63	14.35	9.54–21.15	6.54–32.17	
EGF (pg/mL)	PRP	4.98	0.97	4.85	4.48–5.90	2.98–6.22	<0.0001
	AS	39.06	20.18	34.24	27.72–38.63	19.87–98.22	
Fibronectin (pg/mL)	PRP	929.60	111.50	918.20	833.53–1063.94	778.48–1077.68	=0.0005
	AS	823.64	98.49	780.72	760.12–829.82	703.30–1054.98	
VEGF (pg/mL)	PRP	175.45	65.93	162.50	117.06–216.85	79.45–315.79	<0.0001
	AS	717.35	488.15	524.51	383.36–907.14	235.89–2018.68	
P substance (pg/mL)	PRP	112.58	27.28	115.92	94.54–137.95	69.72–154.92	=0.0125
	AS	127.51	26.56	121.91	116.52–147.58	65.73–163.66	
	AS	127.51	26.56	121.91	116.52–147.58	65.73–163.66	
IGF (pg/mL)	PRP	260.15	74.18	249.10	206.45–301.90	110.30–395.25	=0.1627
	AS	235.05	94.21	242.20	179.55–318.65	77.90–366.90	
	AS	235.05	94.21	242.20	179.55–318.65	77.90–366.90	

**Table 4:** Changes in BCVA and OSDI scores in AS and PRP in the study participants throughout the 3-month observation period by preparation administered.

Analysed Trait	Study Group	Time Point	Statistical Parameter					*p*-Value	
			M	SD	Me	Q_1_–Q3	Min.–Max.		
BCVA logMAR	PRP	Baseline	0.21	0.23	0.19	0.05–0.40	0.00–1.00	=0.0046	=0.0063
		+1 month	0.15	0.18	0.07	0.00–0.22	0.00–0.70		
		+3 months	0.09	0.12	0.02	0.00–0.22	0.00–0.30		
	AS	Baseline	0.06	0.07	0.05	0.00–0.10	0.00–0.22	=0.0010	
		+1 month	0.05	0.05	0.05	0.00–0.10	0.00–0.15		
		+3 months	0.04	0.04	0.05	0.00–0.10	0.00–0.10		
OSDI score	PRP	Baseline	79.00	12.71	75	69.40–88.80	54.16–97.50	<0.0001	=0.0031
		+1 month	65.54	11.62	70	57.99–78.00	50.00–87.00		
		+3 months	59.81	10.37	60	52.27–67.50	45.83–77.00		
	AS	Baseline	79.85	13.12	82.55	72.72–91.00	52.50–95.00	<0.0001	
		+1 month	70.48	12.13	71.59	60.10–81.25	49.00–87.50		
		+3 months	70.59	19.20	70.22	56.26–79.00	47.00–120.00		

**Table 5:** Changes in Schirmer test, oral Schirmer test, and TBUT in AS and PRP in the study participants throughout the 3-month observation period by preparation administered.

Analysed Trait	Study Group	Time Point	Statistical Parameter					*p*-Value	
			M	SD	Me	Q_1_–Q3	Min.–Max.		
Schirmer test (mm)	PRP	Baseline	2.95	2.80	2.00	1–3	0–9	<0.0001	=0.1502
		+1 month	4.09	2.62	3.00	2–5	1–10		
		+3 months	5.00	2.86	4.00	3–7	0–11		
	AS	Baseline	2.91	2.79	2.50	1–4	0–10	=0.0002	
		+1 month	3.59	2.77	3.50	1–5	0–10		
		+3 months	4.00	3.41	4.00	1–6	0–12		
Oral Schirmer test (mm)	PRP	Baseline	2.09	3.04	1	0–4	0–10	=0.0023	NS
		+1 month	2.18	2.99	1	0–4	0–10		
		+3 months	2.23	3.02	1	0–4	0–10		
	AS	Baseline	1.09	1.34	1	0–2	1–4	NS	
		+1 month	1.09	1.34	1	0–2	1–4		
		+3 months	1.09	1.34	1	0–2	1–4		
TBUT (s)	PRP	Baseline	5.364	1.866	5	4–7	2–10	<0.0001	=0.1714
		+1 month	5.682	1.783	6	4–7	3–9		
		+3 months	5.954	2.380	6	4–7	1–10		
	AS	Baseline	4.727	1.804	4	3–6	2–8	<0.0001	
		+1 month	5.545	2.304	5	4–7	2–10		
		+3 months	5.000	2.795	4	3–7	1–11		

**Table 6:** Changes in ocular surface staining and conjunctival hyperaemia in AS and PRP in the study participants throughout the 3-month observation period by preparation administered.

Study Group	Time Point	Grade	PRP		AS		*p*-Value	
			*n*	*%*	*n*	*%*	Repeated Measures	Between-Group
Corneal staining, Oxford scale	Baseline	0					PRP <0.0001 AS <0.0001	0.2513
		1						
		2	6	27.3	7	31.8		
		3	6	27.3	6	27.3		
		4	10	45.4	9	40.9		
		5						
	+1 month	0						
		1			2	9.1		
		2	10	45.5	6	27.3		
		3	9	40.9	7	31.8		
		4	3	13.6	7	31.8		
		5						
	+3 months	0			2	9.1		
		1	3	13.6				
		2	15	68.2	11	50.0		
		3	3	13.6	5	22.7		
		4	1	4.6	4	18.2		
		5						
Conjunctical staining, Oxford scale	Baseline	0					PRP <0.0001 AS <0.0001	0.5120
		1						
		2	8	36.36	6	27.27		
		3	11	50	13	59.09		
		4	2	9.09	3	13.64		
		5	1	4.55	0	0.00		
	+1 month	0						
		1			2	9.09		
		2	15	68.18	10	45.45		
		3	6	27.27	8	36.36		
		4	1	4.55	2	9.09		
		5						
	+3 months	0			2	9.09		
		1	5	22.73	1	4.55		
		2	14	63.64	10	45.45		
		3	2	9.09	5	22.73		
		4	1	4.55	4	18.18		
		5						
Conjunctival hyperaemia	Baseline	0					PRP <0.0001 AS <0.0001	0.1089
		1						
		2	7	31.8	8	36.4		
		3	12	54.6	10	45.4		
		4	3	13.6	4	18.2		
	+1 month	0						
		1			4	18.2		
		2	15	68.2	7	31.8		
		3	6	27.3	9	40.9		
		4	1	4.5	2	9.1		
	+3 months	0						
		1	5	22.7	6	27.3		
		2	17	72.3	8	36.4		
		3			7	31.8		
		4			1	4.5		

**Table 7:** Changes in meibomian gland parameters in AS and PRP in the study participants throughout the 3-month observation period by preparation administered.

Study Group	Time Point	Grade	PRP		AS		*p*-Value	
			*n*	%	*n*	%	Repeated Measures	Between-Group
Quality of Meibomian gland secretion	Baseline	0					PRP NS AS NS	NS
		1	16	72.73	16	72.73		
		2	4	18.18	6	27.27		
		3	2	9.09	6	27.3		
	+1 month	0						
		1	18	81.82	16	72.73		
		2	2	9.09	6	27.3		
		3	2	9.09	0	0		
	+3 months	0						
		1	17	80.95	16	72.73		
		2	2	9.09	6	27.3		
		3	2	9.09	0	0		
Expressibility of Meibomian gland	Baseline	0					PRP NS AS NS	0.1353
		1	16	72.72	18	81.82		
		2	4	18.18	4	18.18		
		3	2	9.09	0	0		
	+1 month	0						
		1	18	81.82	18	81.82		
		2	2	9.09	6	27.3		
		3	2	9.09	0	0		
	+3 months	0						
		1	18	81.82	18	81.82		
		2	2	9.09	4	18.18		
		3	2	9.09	0	0		

## Data Availability

The datasets used and/or analyzed during the current study are available from the corresponding author on reasonable request.
